# A High-Performance Face Illumination Processing Method via Multi-Stage Feature Maps

**DOI:** 10.3390/s20174869

**Published:** 2020-08-28

**Authors:** Shenggui Ling, Ye Lin, Keren Fu, Di You, Peng Cheng

**Affiliations:** 1 National Key Laboratory of Fundamental Science on Synthetic Vision, Sichuan University, Chengdu 610065, China; 2017326040006@stu.scu.edu.cn (S.L.); 2017326040003@stu.scu.edu.cn (Y.L.); 2019326045009@stu.scu.edu.cn (D.Y.); 2 Department of Information Technology, Neijiang Vocational&Technical College, Neijiang 641000, China; 3 College of Computer Science, Sichuan University, Chengdu 610065, China; fkrsuper@scu.edu.cn; 4 School of Aeronautics and Astronautics, Sichuan University, Chengdu 610065, China

**Keywords:** face illumination, face preprocessing, illumination processing, shadow removal, deep learning

## Abstract

In recent years, Generative Adversarial Networks (GANs)-based illumination processing of facial images has made favorable achievements. However, some GANs-based illumination-processing methods only pay attention to the image quality and neglect the recognition accuracy, whereas others only crop partial face area and ignore the challenges to synthesize photographic face, background and hair when the original face image is under extreme illumination (Image under extreme illumination (extreme illumination conditions) means that we cannot see the texture and structure information clearly and most pixel values tend to 0 or 255.). Moreover, the recognition accuracy is low when the faces are under extreme illumination conditions. For these reasons, we present an elaborately designed architecture based on convolutional neural network and GANs for processing the illumination of facial image. We use ResBlock at the down-sampling stage in our encoder and adopt skip connections in our generator. This special design together with our loss can enhance the ability to preserve identity and generate high-quality images. Moreover, we use different convolutional layers of a pre-trained feature network to extract varisized feature maps, and then use these feature maps to compute loss, which is named multi-stage feature maps (MSFM) loss. For the sake of fairly evaluating our method against state-of-the-art models, we use four metrics to estimate the performance of illumination-processing algorithms. A variety of experimental data indicate that our method is superior to the previous models under various illumination challenges in illumination processing. We conduct qualitative and quantitative experiments on two datasets, and the experimental data indicate that our scheme obviously surpasses the state-of-the-art algorithms in image quality and identification accuracy.

## 1. Introduction

**Figure 1:**
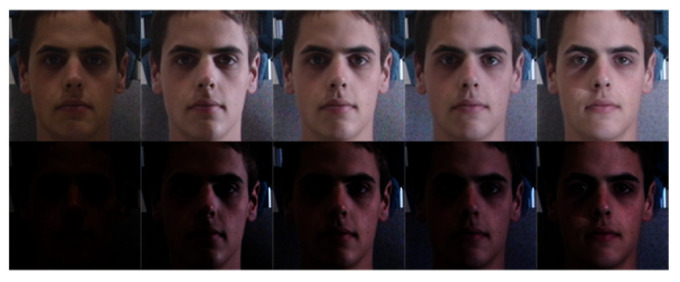
Some poor-lighted faces under various illumination conditions.

As is known to all, the performance of computer vision tasks will degrade when the image sensor is under poor light conditions. As shown in Figure 1, many reasons, such as the excessive exposure and the lack of exposure of the image sensor, the intensity and direction of the light, could make the lighting conditions complicated. Face appearances can change dramatically due to illumination variations [1]. Therefore, illumination processing of facial image under various illumination conditions is highly desired especially in face recognition, expression recognition and so on, due to its wide application in security, health-care, marketing and so on, more and more people are doing research in this field.

In recent decades, in order to solve the illumination problem, experts around the world have come up with various solutions. Most works concentrate on the illumination processing of gray image [2,3,4,5,6,7]. In contrast, research on illumination processing of color image has developed slowly in this field. The data distribution of color image is more complex than that of the gray image is the primary reason to hinder the development of relevant research. As a pioneering work, Faisal et al. [8] combine Phong’s lighting model and a 3D face model to process illumination of color face. Unfortunately, due to the requirement for 3D point clouds and a large amount of computation, this method has limited practical application.

With the developments of hardware and deep learning, illumination processing is gradually evolved from traditional ways to deep learning-based techniques. Ma et al. [9] first use Generative Adversarial Networks (GANs) to process illumination of facial images. Then Ma et al. [10] deal with face illumination by combining triplet loss and GANs. Han et al. [11] put forward asymmetric joint GANs to process facial illumination. Zhang et al. present IL-GAN [12] model based on variational auto-encoder and GANs for processing face illumination.

However, some GANs-based illumination-processing methods only pay attention to the image quality and neglect the recognition accuracy, whereas others only crop partial face area and ignore the challenges to synthesize photographic face, background and hair when the original face image is under extreme illumination conditions. Moreover, the recognition accuracy is low when the face image under extreme illumination conditions. We can use image-to-image translation technique to accomplish the illumination processing of face images. The poor-lighted face images belong to a domain, whereas the standard illumination face images belong to another domain. For these reasons, and inspired by the success of GANs on image-to-image translation, we consider the illumination-processing problem similar to the way of image translation. Our purpose is not only to synthesize photographic face, background and hair when the original face image is under extreme illumination conditions but also preserve identity effectively. The following items are our primary contributions:

1. First, we present a multi-stage feature maps (MSFM) loss that uses different convolutional layers of pre-trained feature network to extract varisized feature maps, and then use these feature maps to compute loss. MSFM loss and our elaborately designed generator are conducive to generating high-quality images and preserving identity effectively.
2. Secondly, our method can effectively synthesize photographic face, background, hair and preserve identity when the original face image is under extreme illumination conditions.
3. Finally, we conduct qualitative and quantitative experiments on two databases and a variety of experimental data indicate that our method significantly surpasses the state-of-the-art methods in image quality and identification accuracy.

We organize our paper as follows. We describe the related work on illumination processing in Section 2. In Section 3, we introduce the proposed method in detail. In Section 4, we show the qualitative and quantitative experimental data. Finally, we draw conclusions in Section 5.

## 2. Related Work

At the beginning of this section, the traditional illumination-processing algorithms are briefly introduced first, and the state-of-the-art GANs such as CycleGAN [13], DMIT [14], EDIT [15], and Pix2Pix [16] that are relevant to our method are in the second part. In the end, we present deep-learning-based illumination-processing methods.

### 2.1. Traditional Illumination-Processing Methods

Over recent decades, numerous works have been put forward for solving the illumination problem. In 1987, Pizer et al. [2] proposed adaptive histogram equalization to enhance image contrast. Afterward, many researchers extend the histogram equalization algorithm. For instance, Shan et al. [17] propose region-based histogram equalization to deal with illumination. Xie et al. [18] put forward block-based histogram equalization for illumination processing. To encode rich information on the edge orientations, Lee et al. [19] present orientated local histogram equalization to compensate illumination.

In 1999, Shashua et al. propose the quotient image method [20] that provides an invariant approach to deal with the illumination. Afterward, many researchers extend the quotient image algorithm. For instance, Shan et al. [17] develop gamma intensity correction by introducing an intensity mapping and quotient image relighting. Wang et al. [21] put forward self-quotient image. Chen et al. [22] produce the TV-based quotient image model for illumination processing. Srisuk et al. [23] propose Gabor quotient image by extending the self-quotient image. An et al. [24] propose a decomposed image under L1 and L2 norm constraint, then obtain illumination invariant large-scale part by region-based histogram equalization and get illumination invariant small-scale part by self-quotient image.

Adini et al. [1] propose logarithmic transformation, directional gray-scale derivation, and Laplacian of Gaussian for illumination processing. Single-scale retinex [25] is put forward by Jobson et al. for processing illumination. W et al. [26] propose Gaussian high pass to process illumination. Local processing technology [4] proposed by Xie et al. can improve the uneven illumination effectively. Chen et al. [27] propose a lighting processing method based on the generic intrinsic illumination subspace. Du et al. [28] present wavelet-based illumination-processing method. Chen et al. [5] propose logarithmic total variation for processing illumination. Chen et al. [3] put forward a new method named logarithmic discrete cosine transformation for illumination compensation and processing. Tan and Triggs [7] process illumination by combining some existing methods such as gamma correction, difference of Gaussian filtering, contrast equalization and masking, which is called TT in the literature [29]. Fan et al. [30] propose a method named homomorphic filtering-based illumination processing. The filter’s key component is a difference of Gaussian.

Wang et al. [31] propose illumination processing based on Weber’s Law. Zhao et al. [32] process illumination by using self-lighting ratio to suppress illumination differences in the frequency domain. A linear representation-based face illumination-processing method is put forward by Li et al. [33]. BimaSenaBayu et al. [34] propose an adaptive contrast ratio based on appearance estimation model and shadow coefficient model. Goel et al. [35] put forward an illumination-processing method based on discrete wavelet transformation and discrete cosine transformation. Vishwakarma [36] proposes a fuzzy filter applied over the low-frequency discrete cosine transformation coefficients method for illumination processing. Zhao et al. [37] use ambient, diffuse, and specular lighting maps to decompose lighting effect and estimate the face albedo. Tu et al. [38] use an energy minimization framework to process illumination. Ahmad et al. [39] use independent component analysis and filtering to process illumination. Zhang et al. [40] use patch-based dictionary learning (DL) to process face illumination. Zheng et al. [41] combine difference of Gaussian filters and difference of bilateral filters for illumination processing. Zhang et al. [42] generate the chromaticity intrinsic image (CII) in a log chromaticity space that is robust to illumination variations by combining Phong’s Model and Lambertian Model. Liu et al. [43]. use fusion-based descattering and color tone correction to enhance the illumination of underwater image.

### 2.2. Illumination-Processing Methods Based on Deep Learning

The developments of machine learning and deep learning accelerate the rapid progress of GANs. The development of GANs [44] brings extraordinary vitality to the image generation. With the help of the combination of GANs and CNN, DCGAN [45] makes a great leap in the ability of image generation. By specifying the input conditions, conditional GAN [46] can generate the specific target photos. With the development of GANs, image translation has also achieved impressive development. Isola et al. [16] propose Pix2Pix for a wide range of supervised domain translation tasks. Since obtaining a mass of paired data is not easy and impractical for many domain translation tasks, DualGAN [47], DiscoGAN [48], CycleGAN [13], DMIT [14] and EDIT [15] are proposed to learn two cross-domain translation models that obey the cycle consistent rule from unpaired data.

Recently, illumination-processing of image based on deep learning has made favorable achievements. Ma et al. [49] use deep convolutional neural network and HSI color space to enhance low-light image. Ma et al. [9] first use Generative Adversarial Nets to process illumination of facial image. Then Ma et al. [10] process face illumination by combining triplet loss and GANs. Han et al. [11] put forward asymmetric joint GANs to process facial illumination. Their method contains two GANs, one of which is employed to process illumination, the other is to maintain personalized facial structures. Zhang et al. put forward IL-GAN [12] based on variational auto-encoder and GANs for processing face illumination. AJGAN [11] shows that it is difficult to obtain a favorable illumination-processing result by unsupervised illumination-processing methods. Therefore, we still use weakly supervised method to process illumination of color and gray faces in this paper.

Although the above illumination-processing methods can deal with illumination effectively and preserve identity well, there are some problems. For example, Ma et al. [9,10] only pay attention to image quality and neglect recognition accuracy. AJGAN [11] and IL-GAN [12] not only conduct face recognition experiments but also illustrate preferable image quality, but their methods only crop partial face area and ignore the challenges to synthesize photographic face, background and hair when the original face image is under extreme illumination conditions. Moreover, the face recognition methods in their paper are outmoded. The main problem is that the recognition accuracy is low when the faces under extreme illumination conditions. For these reasons, we put a new scheme to process the illumination of color and gray faces. We use advanced ResNet-50 [50] pre-trained on VGGFace2 dataset [51], Light-CNN-9 and Light-CNN-29 [52] pre-trained on CASIA-WebFace and MS-Celeb-1M dataset for face identification and the structural similarity (SSIM) [53] index, the visual information fidelity (VIF) [54] and the feature similarity (FSIM) [55] to evaluate our method comprehensively.

## 3. Proposed Method

We define *X* as poor-lighted faces and *Y* as standard illumination faces. Given sets $x_{ij}\in X$, $y_{i}\in Y$, *i* denotes identity and *j* means light type. We expect $H\left(G\left(x_{ij}\right)\right)=H\left(y_{i}\right)$, which means that after translating various light into a standard one, the synthesized face images $G(x_{ij})$ and the corresponding standard illumination $y_{i}$ have the same identity *i*. Identity preservation is very important in various image-to-image translation about face images [56]. In this paper, *H* means feature extractor such as ResNet-50, Light-CNN-9 and Light-CNN-29. Next, we denote $x_{ij}$ and $y_{i}$ with *x*, *y* for short.

### 3.1. Overall Framework

**Figure 2:**
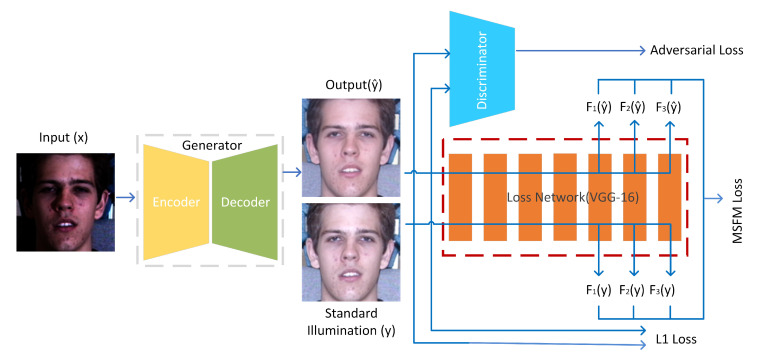
Our overall network framework. The core of our method mainly contains 3 parts: an elaborately designed generator, a discriminator and a loss network.

At the beginning of this section, we first present our illumination-processing method of face image in detail. Figure 2 shows the block diagram of the proposed method, which takes a set of poor-lighted face images as input and outputs a set of well-lighted face images in an end-to-end way. From the Figure 2, we can learn that the core of our approach is made up of a generator, a loss network for extracting varisized feature maps and using these feature maps to compute multi-stage feature maps (MSFM) loss. An encoder and a decoder make up our generator together. In the testing phase, we just use the generator to transform poor-lighted face images into well-lighted images. We use 3 loss items: adversarial loss, multi-stage feature maps (MSFM) loss and L1 distance loss. *F* is introduced in detail when we narrate multi-stage feature maps (MSFM) loss in Section 3.3.

### 3.2. Generator and Discriminator Architecture

**Figure 3:**
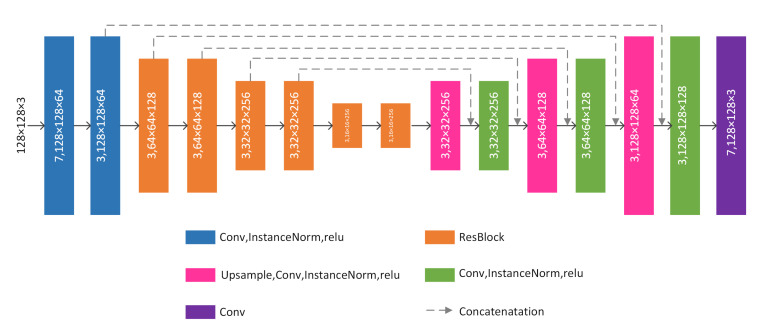
The detailed structure of our generator. 7, 128 ×128×64 means kernel_size=7, feature maps with 128 values in width, 128 values in height and 64 channels.

The generator of our method is inspired by the components of residual network [50] and U-net [57]. Our generator consists of 9 convolutional layers, 6 residual blocks and 3 up-sample layers, each of which is equipped with a ReLU as activation. Details of the generator are illustrated in Figure 3. From the Figure 3, we can know that the input size of the generator is designed to be a 128×128 color image. The output resolution of our generator is 128×128 pixels in size. The dotted lines in Figure 3 are skip connections that are conducive to feature retention. In the middle 6 convolutional layers, we use dropout to avoid over-fitting and special up-sample blocks in the decoder of our generator for enhancing the synthetic ability of our model. For this special design, which further enhances the ability of feature retention. Because InstanceNorm [58] has the characteristics of preventing instance-specific mean and covariance shift simplifying the learning process, we use InstanceNorm after each convolutional layers. InstanceNorm can be computed by:

(1) $$y_{tijk}=\frac{x_{tijk}-\mu_{ti}}{\sqrt{\sigma_{ti}^{2}+\varepsilon }},$$

(2) $$\mu_{ti}=\frac{1}{HW}\sum_{l=1}^{W}\sum_{m=1}^{H}x_{tilm},$$

(3) $$\sigma_{ti}^{2}=\frac{1}{HW}\sum_{l=1}^{W}\sum_{m=1}^{H}{\left(x_{tilm}-\mu_{ti}\right)}^{2},$$

where $x\in R^{T\times C\times W\times H}$ is a tensor including *T* images. $x_{tijk}$ mean its tijk-th element, *k* and *j* are spatial dimensions, *i* denotes color channel, *t* is the index of the image in the batch.

**Figure 4:**
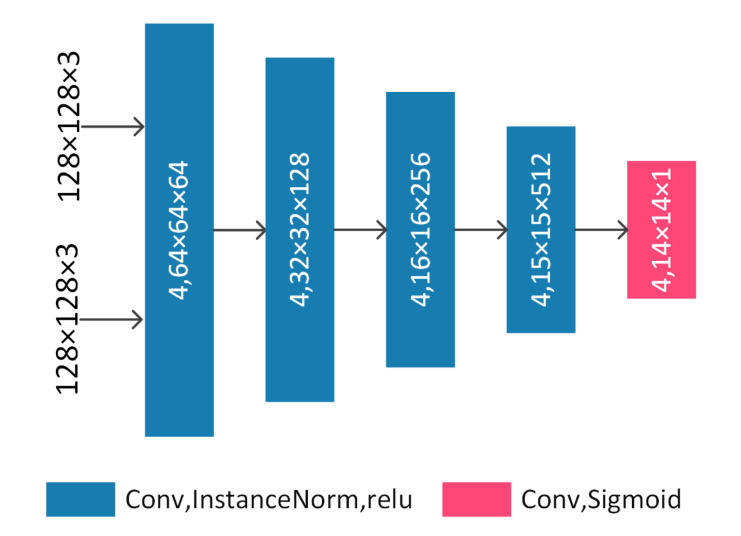
The detailed structure of our discriminator. The input images are a 128×128 paired color images such as (x,y) and (x,G(x)). 4, 64 ×64×64 means kernel_size=4, feature maps with 64 values in width, 64 values in height and 64 channels.

The discriminator of our method is inspired by the components of Pix2Pix [16]. The detailed structure of our discriminator is shown in Figure 4. The ReLU is used as activation after the left four convolutional layers and we replace BatchNorm with InstanceNorm. The input size of the discriminator is designed to be a 128×128 paired color images such as (x,y) and (x,G(x)). We use InstanceNorm after the left four convolutional layers.

### 3.3. Objective Function

Three terms make up our objective function together: a multi-stage feature maps loss for making the ground truth and the generative results more similar, an adversarial loss for making the real distribution and the distribution of synthesized images more similar. A L1 distance loss for improving the performance of our method further.

**Adversarial Loss:** The adversarial process is made up of the generator *G* and the discriminator *D*. *D* attempts to discriminate the generated fake image *G*(*x*) and the ground truth image *y* whereas *G* strives to generate fake image *G*(*x*) to fool discriminator *D*. The objective function is as follows:

(4) $$\begin{array}{cc} L_{GAN}\left(G,D,x,y\right) & =E_{y∼P_{Y}}\left[logD\left(y\right)\right]\\ \; & +\;E_{x∼P_{X}}\left[log\left(1-D\left(G\left(x\right)\right)\right)\right], \end{array}$$

where *x* denotes input image (poor-lighted face), whereas *y* is target image (standard illumination).

**Multi-Stage Feature Maps Loss:** Experimental data indicate that early layers of loss network *F* (VGG-16) pre-trained on the ImageNet dataset [59] tend to produce smooth facial images, whereas the content and the overall spatial structure can be preserved by the higher layers of *F*, but the color, the texture and the exact shape are not preserved effectively. Therefore, we choose middle layers of *F* as our loss network for synthesizing high-quality facial images.

As narrated before, we hope the synthesized image G(x) and its ground truth *y* to be similar in illumination and to have same identity features. Meanwhile, the generator is not only to generate a well-lighted face image but also to fool the discriminator as soon as possible. We use different layers of pre-trained feature network for extracting varisized feature maps, and then use these feature maps to compute feature loss, named multi-stage feature maps (MSFM) loss. The multi-stage feature maps (MSFM) loss can be computed by:

(5) $$\begin{array}{cc} L_{MSFM}\left(G,x,y\right) & =\sum_{i=1}^{3}\lambda_{i}\times \left|\right|F_{i}\left(y\right)-F_{i}\left(G\left(x\right)\right){\left|\right|}_{2}^{2}\\ \; & =\sum_{i=1}^{3}\lambda_{i}\times \left|\right|F_{i}\left(y\right)-F_{i}\left(\hat{y}\right){\left|\right|}_{2}^{2}, \end{array}$$

*F* is the VGG-16 network that has 13 convolutional layers. $F_{1}$ means 1 to 5 convolutional layers of VGG-16 [60], $F_{2}$ means 1 to 6 convolutional layers from of VGG-16, $F_{3}$ means 1 to 7 convolutional layers of VGG-16. $\lambda_{i}$ is weight parameter, $\hat{y}$ means the output result of our generator, whereas *y* is target image (standard illumination). $F_{1}\left(y\right)$ means that we use the 1 to 5 convolutional layers of VGG-16 to obtain the feature maps of *y*.

**L1 Distance Loss:** Although MSFM loss and adversarial loss make our method gain favorable performance in image quality and recognition accuracy, for the sake of improving the performance of our method further, we add L1 distance loss to our loss function. Because L2 result in blurry synthesized image [16], we choose L1 instead of L2. In this paper, L1 is used to compute the sum of absolute difference between the ground truth image *y* and the generated image $\hat{y}$. L1 distance can be computed by:

(6) $$\begin{array}{cc} L_{L1}\left(G,x,y\right) & =||y-{G\left(x\right)||}_{1}\\ \; & =||y-\hat{y}{\left|\right|}_{1}, \end{array}$$

where *y* is the ground truth, and $\hat{y}$ is the synthesized result.

The final objective function is:

(7) $$\begin{array}{cc} L(G,D,x,y) & =L_{GAN}\left(G,D,x,y\right)\\ \; & +\;L_{MSFM}\left(G,x,y\right)\\ \; & +\;\alpha \times L_{L1}\left(G,x,y\right), \end{array}$$

where α is weight parameter.

## 4. Experimental Results

In this section, the datasets, the implementation details and the qualitative and quantitative results of our algorithm are illustrated in detail. Our experiments are conducted on two sets, one is color set, the other is gray set. We compare with the state-of-the-art unsupervised and supervised deep learning methods on the color set. On the gray set, we mainly make comparison between classical illumination-processing methods and ours.

### 4.1. Datasets

In our paper, we first perform the experiments on the MultiPIE [61] database and then make a comparison with some state-of-the-art methods such as CycleGAN [13], DMIT [14], EDIT [15], Pix2Pix [16], and then compare with the classical methods such as LDCT [3], LN [4], LTV [5], SQI [6], TT [7] and famous CycleGAN [13], Pix2Pix [16] and ours on the Extended YaleB datasets. Finally, we randomly choose some face images from FRGC [62] dataset to verify the generalization performance of our algorithm.

**MultiPIE database:** The MultiPIE [61] database has been extensively used in illumination processing and face recognition. We choose frontal faces, 20 illumination conditions and natural expression, without glasses from session 1 of MultiPIE as our dataset. We detect and crop faces from the dataset with single shot scale-invariant face detector (S3FD) [63] and resize to 128 × 128 as our training and test set. The 07 illumination faces are chosen as standard faces (standard illumination) and the rest are selected as poor-lighted facial images. In the training dataset, 99 individuals are chosen. For making the experiments more challenging, we use gamma correction to make the image become darker. When gamma=2, we think it is not challenging enough. When gamma=4, the mean value of some faces is 0.47 and the max pixel value is 9. The zero values are more than three quarters. Due to the lack of effective information, it is difficult to restore meaningful images. So, we set gamma=3 and we use gamma correction to process all the images, which is not only challenging but also being able to restore meaningful images. In actual application, we can set more gamma values such as 1.1, 1.2 … and so on for obtaining more training data and improving the performance of the method. Thus, the training set has 99×19×2+99=3861 images. The test set has 30 individuals and 30×19×2=1140 images. We use 30 individuals under 38 lighting conditions (19 original lighting conditions and 19 analog lighting conditions obtained from the former’s images by conducting gamma correction when gamma=3) to test CycleGAN, DMIT, EDIT, Pix2Pix and our method.

**Extended YaleB:** The Extended YaleB that has 38 subjects under 64 illumination conditions is widely used to evaluate different illumination-processing methods. We divide this dataset according to the literature [64,65], 1 to 28 (1792 images) are used to train all the deep learning-based approaches, 29 to 38 (630 images) are used for test. Next, we call it YaleB for short.

**FRGC v2 dataset:** The Face Recognition Grand Challenge (FRGC) [62] dataset contains 3D images and high resolution controlled and uncontrolled stills under various illumination conditions. We randomly choose some 2D faces to verify the generalization performance of our algorithm. Next, we call it FRGC for short.

### 4.2. Implementation Details

As to the encoder and decoder, we use ReLU for activation first. For gradient descent, we use Adam [66] optimizer, and choose a learning rate of 0.0002 with momentum parameters $\beta_{1}$ = 0.95, $\beta_{2}$ = 0.999. We set batchsize=8 during training period. 100 epochs have completed within about 200 min on the MultiPIE dataset. Moreover, during the training period, we use random cropping for data enhancement. In this work, we set α=0.1. By setting different values for $\lambda_{1}$, $\lambda_{2}$ and $\lambda_{3}$, we get 4 combinations of loss items. We set $\lambda_{1}$ = 1.0, $\lambda_{2}$ = 0.0 and $\lambda_{3}$ = 0.0 to train a model using 1 to 5 convolutional layers of VGG-16 for extracting feature maps and computing loss. We choose $\lambda_{1}$ = 0.0, $\lambda_{2}$ = 1.0 and $\lambda_{3}$ = 0.0 to train a model using 1 to 6 convolutional layers of VGG-16 for extracting feature maps and computing loss. We set $\lambda_{1}$ = 0.0, $\lambda_{2}$ = 0.0 and $\lambda_{3}$ = 1.0 to train a model using 1 to 7 convolutional layers of VGG-16 for extracting feature maps and computing loss. For balancing the image quality and identity feature, we set $\lambda_{1}$ = 0.91, $\lambda_{2}$ = 0.08 and $\lambda_{3}$ = 0.01 and train a model using multi-stage feature maps loss.

### 4.3. Metrics

At present, most literature evaluates the performance of illumination-processing algorithms from two aspects: one is to compare recognition accuracy, the other is to illustrate some face images before and after processing by various methods. We make use of cosine similarity of feature vectors for face recognition. For the purpose of more comprehensively estimating the performance of various illumination-processing algorithms, except for comparing recognition accuracy and illustrating examples, we also adopt the structural similarity (SSIM) [53] index, visual information fidelity (VIF) [54] and the feature similarity (FSIM) [55] to evaluate the performance of various illumination-processing methods. Cosine similarity, VIF, SSIM and FSIM are briefly introduced as follows:

1. The cosine similarity is a metric to determine how similar the two vectors are. It computes the cosine of the angle between two vectors. The smaller the angle is, the higher similarity is. The cosine similarity is computed by:
  (8) $$\cos\left(\theta \right)=\frac{\sum_{i=1}^{n}A_{i}\times B_{i}}{\sqrt{\sum_{i=1}^{n}{\left(A_{i}\right)}^{2}}\times \sqrt{\sum_{i=1}^{n}{\left(B_{i}\right)}^{2}}},$$
  where *A* and *B* are the feature vectors which are extracted by the ResNet-50 [50] pre-trained on VGGFace2 [51], the Light-CNN-9 [52] and the Light-CNN-29 [52] pre-trained on CASIA-WebFace and MS-Celeb-1M. $A_{i}$ denotes i-th element of vector *A*, $B_{i}$ denotes i-th element of vector *B*.
2. The visual information fidelity (VIF) [54] is a metric to measure the information fidelity between the ground truth image and the generated image. *VIF* is defined as:
  (9) $$VIF=\frac{\sum_{j\in subbands}I\left({\vec{C}}^{N,j};{\vec{F}}^{N,j}|S^{N,j}\right)}{\sum_{j\in subbands}I\left({\vec{C}}^{N,j};{\vec{E}}^{N,j}|S^{N,j}\right)},$$
  where $I({\vec{C}}^{N,j};{\vec{F}}^{N,j}|S^{N,j})$ and $I({\vec{C}}^{N,j};{\vec{E}}^{N,j}|S^{N,j})$ denote the information that can ideally be extracted by the brain from a particular sub-band in the ground truth image and the generated image respectively.
3. The structural similarity (SSIM) [53] is a widely used metric to measure the level of similarity in structure between the ground truth and the synthesized image. The two images are exactly equal when the SSIM values of two images is equal to 1. *SSIM* is defined as:
  (10) $$SSIM\left(x,y\right)=\frac{\left(2\mu_{x}\mu_{y}+C_{1}\right)\left(2\sigma_{xy}+C_{2}\right)}{\left(\mu_{x}^{2}+\mu_{y}^{2}+C_{1}\right)\left(\sigma_{x}^{2}+\sigma_{y}^{2}+C_{2}\right)},$$
  where *x* and *y* means two images, $\mu_{x}$ is the mean value of image *x*, $\mu_{y}$ is the mean value of image *y*. $\sigma_{x}$ and $\sigma_{y}$ are standard variances of image *x* and *y*. $C_{1}$ and $C_{2}$ are two constants.
4. The feature similarity (FSIM) [55] is a widely used metric to measure the feature similarity between the ground truth and the synthesized image. The closer the FSIM value is to 1, the more similar feature the two images have. *FSIM* is defined as:
  (11) $$FSIM=\frac{\sum_{x\in \Omega }S_{L}\left(x\right)PC_{m}\left(x\right)}{\sum_{x\in \Omega }PC_{m}\left(x\right)},$$
  where Ω is the whole image spatial domain, $PC_{m}\left(x\right)$ is the phase congruency of image *x*, and $S_{L}\left(x\right)$ is the overall similarity of two images.

In summary, the closer the values of SSIM, VIF and FSIM are to 1, the higher quality the image.

### 4.4. Qualitative Comparisons

**Figure 5:**
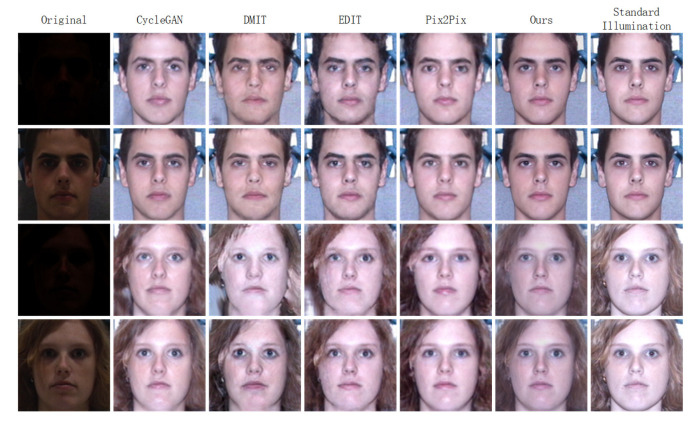
Comparisons between the CycleGAN [13], the DMIT [14], the EDIT [15], the Pix2Pix [16] and ours on the MultiPIE database. The first column: original poor-lighted face images. The second to sixth columns: synthesized results of the original poor-lighted face images.

In this section, we illustrate some poor-lighted faces processed by CycleGAN [13], DMIT [14], EDIT [15], Pix2Pix [16] and our method. As can be observed from the Figure 5, in general, all these methods can process various illumination conditions and obtain better visual effects, but some methods have drawbacks. As illustrated in Figure 5, the first column is the original poor-lighted faces under various illumination conditions. From the second to the sixth columns are faces processed by CycleGAN, DMIT, EDIT, Pix2Pix and ours. From the second column, we can see that CycleGAN obtains favorable face images, but the first and third faces are not photo-realistic enough, moreover, the background of the first faces has some shadow and the hair of the third faces is translated into background. From the third and the fourth columns of Figure 5, we can see clearly that DMIT and EDIT cannot discriminate background and hair well. Although Pix2Pix obtains preferable faces, its outputs have some blemishes. For instance, the first face of the fifth column is not photo-realistic and natural enough, moreover, from the third and fourth faces, we can know that some hair areas are not bright enough. From the sixth column of Figure 5, we can learn that our method cannot only translate poor-lighted faces under various illumination conditions into well-lighted and photo-realistic faces but also discriminate background and hair well.

**Figure 6:**
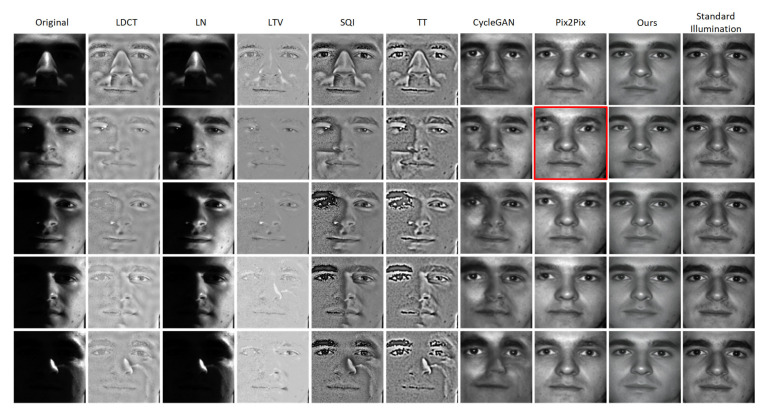
Comparisons between the LDCT [3], the LN [4], the LTV [5], the SQI [6], the TT [7], the CycleGAN [13], the Pix2Pix [16] and ours on the YaleB database. The first column: original poor-lighted face images. The second to ninth columns: synthesized results of the original poor-lighted face images.

Figure 6 illustrates the illumination-processing results of the eight methods on the YaleB database. The synthesized faces of LDCT [3] are blurry. The output results of LN [4] are still dark and have many shadows. The LTV [5] method obtains smooth synthesized face images. The SQI [6] and TT [7] methods get noisy output results and cannot process cast shadows well. From the seventh column of Figure 6, we can see the faces generated by CycleGAN [13] are not natural and some face area is too bright. At the eighth column of Figure 6, we can see that some faces synthesized by Pix2Pix [16] have 3 eyes and some faces are distorted. Our method can not only process illumination effectively but also obtain high-quality synthesized face images with good visual effects. In the next section, we will evaluate all the aforementioned methods with four metrics objectively.

### 4.5. Quantitative Evaluation

Next, we conduct face identification and full reference image quality assessment (FR-IQA) on the MultiPIE database and the YaleB database separately.

#### 4.5.1. Quantitative Evaluation on the MultiPIE Database

**Figure 7:**
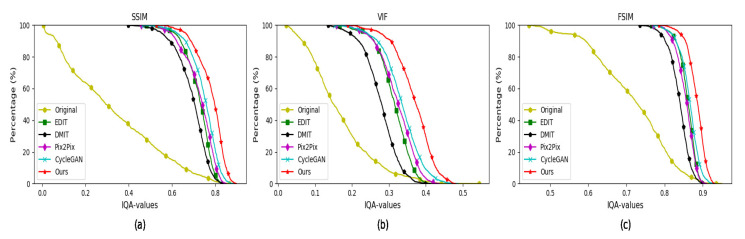
FR-IQA values of the face images synthesized by various algorithms on the MultiPIE dataset. (**a**) SSIM values of generated faces; (**b**) VIF values of synthesized faces; (**c**) FSIM values of synthesized faces.

As to FR-IQA, we conduct experiments on the MultiPIE database. When we compute the FR-IQA of the synthesized faces, the faces with standard illumination are chose as reference images. We compute FR-IQA by three metrics: (1) the first is the structural similarity (SSIM) index [53]; (2) the second is the visual information fidelity (VIF) index [54]; (3) the third is the feature similarity (FSIM) [55]. The FR-IQA values of the faces synthesized by CycleGAN, Pix2Pix, EDIT, DMIT and our method are shown in Figure 7, which shows the higher FR-IQA values are, the higher percentage of images on the horizontal axis is. It is obvious that our algorithm obtains higher FR-IQA values than the other four approaches. In Table 1, we illustrate the average FR-IQA of various algorithms, which demonstrates that our method is superior to the others.

As shown in Table 2, we use ResNet-50 [50] pre-trained on VGGFace2 [51], Light-CNN-9 [52] and Light-CNN-29 [52] pre-trained on CASIA-WebFace and MS-Celeb-1M to evaluate the recognition accuracy of original poor-lighted face images and faces processed by CycleGAN [13], DMIT [14], EDIT [15], Pix2Pix [16] and our method. From the second column of Table 2, we can know when we use ResNet-50 to recognize the original poor-lighted faces, the recognition accuracy is only 93.86%. After processing by CycleGAN, the recognition accuracy is 98.51% and improved by 4.65%. After processing by DMIT, the recognition accuracy is 93.77% and decreased by 0.09%. After processing by EDIT, its recognition accuracy is 98.25% and improved by 4.39%. After processing by Pix2Pix, the recognition accuracy is 97.89% and improved by 4.03%. After processing by our method, the recognition accuracy is 99.91% and improved by 6.05%. From the third column of Table 2, we can learn that the identification accuracy is improved by 5.26% when we use Light-CNN 9 to recognize the faces processed by our method, whereas CycleGAN, DMIT, EDIT and Pix2Pix only improved by 3.69%, 3.16%, 4.12% and 3.33% separately. From the fourth column of Table 2, we can know that the identification accuracy is improved by 3.33%, 2.72%, 3.51% and 3.16% separately when we use Light-CNN 29 to recognize the faces processed by CycleGAN, DMIT, EDIT and Pix2Pix, whereas our method improves the recognition accuracy by 3.95% and reaches to 100%. It is obvious that recognition accuracy of original poor-lighted faces processed by these methods listed in the table has been improved more less. When we use ResNet-50 to recognize, except for DMIT, all other algorithms improve the recognition accuracy obviously. Compared to other method listed in Table 2, our approach obtains the maximal recognition accuracy.

**Figure 8:**
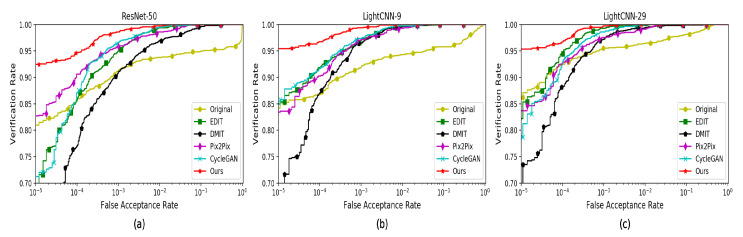
The ROC curves of face verification using ResNet-50, Light-CNN-9 and Light-CNN-29 on the MultiPIE dataset. (**a**) ROC curves of ResNet-50; (**b**) ROC curves of Light-CNN-9; (**c**) ROC curves of Light-CNN-29.

For the sake of further evaluating the performance of the above methods, we also draw ROC curves. As shown in Figure 8, the figure illustrates the Verification Rate (VR) and False Acceptance Rate (FAR). In Table 2, we show the results of top-1 and VR@FAR = 0.1%. From the Figure 8 and Table 2, we can see that our method is better than the others in identity preservation.

#### 4.5.2. Quantitative Evaluation on the YaleB Database

**Figure 9:**
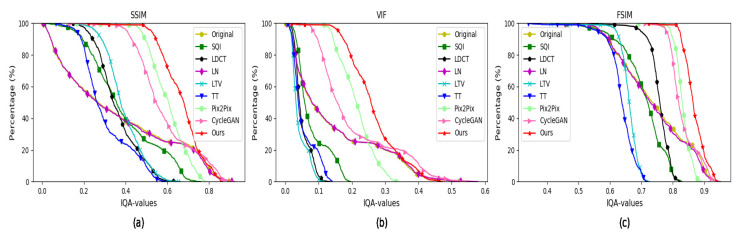
FR-IQA values of the face images synthesized by various algorithms on the YaleB dataset. (**a**) SSIM values of synthesized faces; (**b**) VIF values of synthesized faces; (**c**) FSIM values of synthesized faces.

We choose the standard illumination faces as reference images when we conduct full reference image quality assessment (FR-IQA) experiments on the YaleB database. We use three full reference image quality assessment (FR-IQA) metrics to evaluate the performance of the classical illumination-processing methods and ours: (1) the first is the structural similarity (SSIM) index [53]; (2) the second is the visual information fidelity (VIF) index [54]; (3) the third is the feature similarity (FSIM) [55]. The FR-IQA values of the faces synthesized by LDCT [3], LN [4], LTV [5], SQI [6], TT [7] and our algorithm are shown in Figure 9, which shows the higher FR-IQA values are, the higher percentage of images on the horizontal axis is. Except for a few of our synthesized images are worse than CycleGAN, Pix2Pix in image quality. It is obvious that our algorithm obtains higher average FR-IQA values than the other seven approaches. In Table 3, we illustrate the average FR-IQA of various algorithms, which demonstrates that our method is superior to the others on average FR-IQA values on the YaleB database.

Table 4 shows the top-1 identification accuracy and verification accuracy at 0.1% FAR on the YaleB database using various feature extractor. In the second column of Table 4, we can know that the top-1 identification accuracy of original poor-lighted face images is only 51.26%. The identification rates of the 5 classical illumination-processing methods and Pix2Pix are lower than the original poor-lighted face images. The foremost reason is that the illumination of the face images from the YaleB dataset is too dire. The dire illumination causes the images generated by LDCT, LTV, TT to loss details and to become smooth. As shown in the red box of Figure 6, the verification accuracy of Pix2Pix decreases significantly due to the poor identity retention and distorted faces caused by the extreme illumination conditions. However, our method obtains 89.52% identification rate. From the fourth column of Table 4, we can see that the identification rate reaches 94.29% after illumination processing by our method then using Light-CNN-29 to identify. However, the identification rates of state-of-the-art algorithms such as IL-GAN [12] and AJGAN [11] are 89.61% and 87.20% separately.

**Figure 10:**
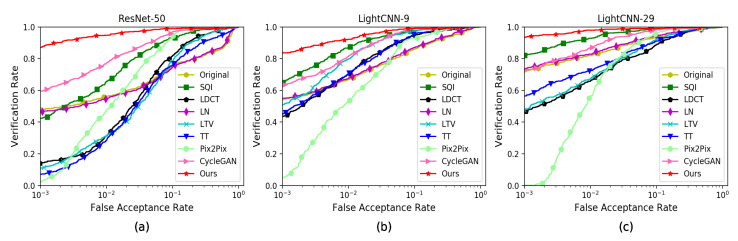
The ROC curves of face verification using ResNet-50, Light-CNN-9 and Light-CNN-29 on the YaleB dataset. (**a**) ROC curves of ResNet-50; (**b**) ROC curves of Light-CNN-9; (**c**) ROC curves of Light-CNN-29.

For the sake of further evaluating the performance of the above methods, we also draw ROC curves. As shown in Figure 10, the figure illustrates the Verification Rate (VR) and False Acceptance Rate (FAR). In Table 4, we show the results of top-1 and VR@FAR = 0.1%. From the Figure 10 and Table 4, we can see that our algorithm precedes the others in identity preservation.

### 4.6. Application to Unseen Dataset

**Figure 11:**
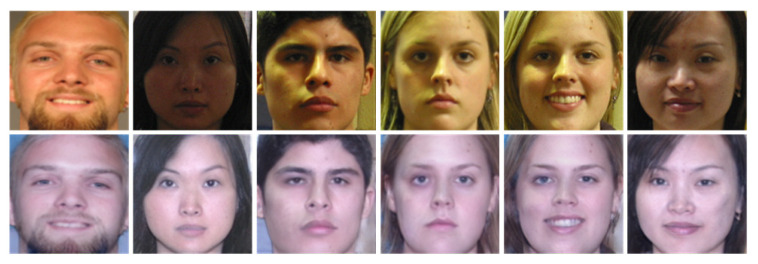
Some poor-lighted faces of FRGC dataset before and after processing by our algorithm. The first row is original facial images under various illumination conditions. The second row is the illumination-processing results of our method.

For the sake of verifying the generalization performance of our method further, we perform illumination-processing experiment on the facial images of FRGC dataset. As illustrated in Figure 11, we can see that the faces in the first row are under various illumination conditions, the second row is the synthesized face images of the first row by our algorithm. It is obvious that our algorithm can not only improve the illumination of faces under various poor-lighted conditions but also keep corresponding identities effectively.

### 4.7. Ablation Studies

Theoretically, different layer of deep convolution network can extract features of different granularity. Inspired by this, we use different layers of VGG-16 for extracting varisized feature maps, and then use these feature maps to compute multi-stage feature maps loss.

As shown in Table 5, we demonstrate the SSIM, VIF and FSIM score of face images processed by our method trained under various loss items. In the first row, conv1-5 means we use 1 to 5 convolutional layers of VGG-16 for extracting feature and computing loss, conv1-6 means we use 1 to 6 convolutional layers of VGG-16 for extracting feature and computing loss, conv1-7 means we use 1 to 7 convolutional layers of VGG-16 for extracting feature and computing loss, combination means to combine conv1-5, conv1-6 and conv1-7 for computing multi-stage feature maps loss. For convenience, we use loss A, B, C, D to indicate the aforementioned 4 losses. Although our method trained under loss A, B and C can obtain high SSIM, VIF and FSIM separately, we increase them by combining loss A, B and C further.

**Figure 12:**
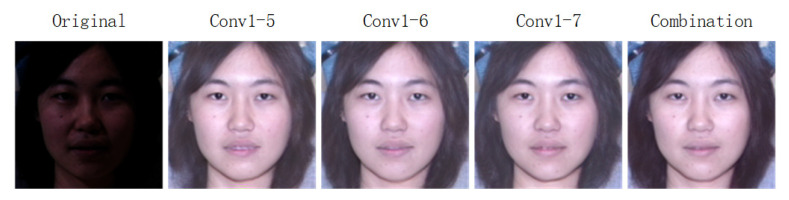
The synthesized results of our method trained under various loss items. Conv1-5 means to use 1 to 5 convolutional layers of VGG-16 for extracting feature and computing loss. Conv1-6 means to use 1 to 6 convolutional layers of VGG-16 for extracting feature and computing loss. Conv1-7 means to use 1 to 7 convolutional layers of VGG-16 for extracting feature and computing loss. Combination denotes to combine the former three loss items for extracting multi-stage feature maps and computing multi-stage feature maps loss.

As illustrated in Figure 12, we illustrate some facial images processed by our method trained under various loss items. The first poor-lighted image is the original image. The second one is obtained by our method trained under 1 to 5 convolutional layers of VGG-16 for extracting feature and computing loss. The third one is generated by our method trained under 1 to 6 convolutional layers of VGG-16 for extracting feature and computing loss. The fourth one is obtained by our method trained under 1 to 7 convolutional layers of VGG-16 for extracting feature and computing loss. Although the former 3 schemes can process illumination effectively and preserve identifies well, the combination scheme can improve the image quality further.

## 5. Conclusions

In this work, we use multi-stage feature maps (MSFM) loss and an elaborately designed architecture based on convolutional neural network and GANs for illumination processing of face images. Our method can synthesize photographic face, background and hair when the original face image is under extreme illumination conditions. Furthermore, our method can not only process illumination of face image under extreme illumination conditions, but also preserve identity information effectively. We discover that face images with favorable quality does not guarantee high recognition accuracy, only the face images that preserve identity features and structure well can obtain high recognition accuracy. Our method will fail when there is no clear boundaries between the clothes and the background, specifically when the background and clothes have the same color, and when the mean value is less than 1 and most pixel values are equal to zero. Although our illumination-processing algorithm achieves preferable results, there is a lot of future work and potential applications here worth continuing to study:

1. To improve our network structure for preserving more texture details.
2. To train a feature extractor and classifier for the facial images after illumination processing by our method.
3. Using our method to process the illumination of other images, such as landscape and medical images.
4. Applying our method to the preprocessing stage of other visual analysis tasks, such as face detection, head pose estimation, facial landmark detection and face alignment, to improve the performance in these tasks.

## Figures and Tables

**Table 1:** Average FR-IQA values of the face images synthesized by various illumination-processing methods on the MultiPIE dataset.

	Metrics	SSIM ↑	VIF ↑	FSIM ↑
Methods				
Original		0.3289	0.1669	0.7165
CycleGAN [13]		0.7477	0.3340	0.8644
DMIT [14]		0.7032	0.2845	0.8446
EDIT [15]		0.7216	0.3132	0.8591
Pix2Pix [16]		0.7265	0.3221	0.8548
Ours		**0.7899**	**0.3788**	**0.8865**

**Table 2:** Top-1 identification accuracy and verification accuracy at 0.1% FAR on the MultiPIE dataset using ResNet-50, Light-CNN-9 and Light-CNN-29.

FR		Top-1			VR@FAR = 0.1%		
	Verifiers	ResNet-50 [50]	Light-CNN-9 [52]	Light-CNN-29 [52]	ResNet-50 [50]	Light-CNN-9 [52]	Light-CNN-29 [52]
Methods							
Original		0.9386	0.9456	0.9605	0.9122	0.9184	0.9552
CycleGAN [13]		0.9851	0.9825	0.9938	0.9666	0.9736	0.9859
DMIT [14]		0.9377	0.9772	0.9877	0.9114	0.9649	0.9728
EDIT [15]		0.9825	0.9868	0.9956	0.9500	0.9701	0.9929
Pix2Pix [16]		0.9789	0.9789	0.9921	0.9587	0.9719	0.9754
Ours		**0.9983**	**0.9991**	**100.000**	**0.9859**	**0.9938**	**0.9974**

**Table 3:** Average FR-IQA values of the face images synthesized by various illumination-processing methods on the YaleB dataset.

	Metrics	SSIM ↑	VIF ↑	FSIM ↑
Methods				
Original		0.3569	0.1413	0.7410
LDCT [3]		0.3623	0.0503	0.7370
LN [4]		0.3526	0.1435	0.7370
LTV [5]		0.3914	0.0409	0.6602
SQI [6]		0.3891	0.0775	0.7092
TT [7]		0.3036	0.0533	0.6365
CycleGAN [13]		0.5849	0.2033	0.8318
Pix2Pix [16]		0.6031	0.2128	0.8316
Ours		**0.6946**	**0.2621**	**0.8660**

**Table 4:** Top-1 identification accuracy and verification accuracy at 0.1% FAR on the YaleB dataset using ResNet-50, Light-CNN-9 and Light-CNN-29.

FR		Top-1			VR@FAR = 0.1%		
	Verifiers	ResNet-50 [50]	Light-CNN-9 [52]	Light-CNN-29 [52]	ResNet-50 [50]	Light-CNN-9 [52]	Light-CNN-29 [52]
Methods							
Original		0.5126	0.7302	0.8064	0.4285	0.4587	0.6492
SQI [6]		0.5032	0.8317	0.9000	0.3031	0.5063	0.6952
LDCT [3]		0.1523	0.5904	0.5889	0.0714	0.3047	0.3571
LN [4]		0.4968	0.7333	0.8206	0.4238	0.4539	0.6650
LTV [5]		0.1317	0.6667	0.5635	0.0317	0.3349	0.3253
TT [7]		0.1064	0.5873	0.6762	0.0301	0.2603	0.4269
CycleGAN [13]		0.6889	0.7762	0.8015	0.5968	0.6333	0.7285
Pix2Pix [16]		0.1889	0.2016	0.1048	0.0222	0.0492	0.0016
Ours		**0.8952**	**0.9016**	**0.9429**	**0.8714**	**0.8746**	**0.9317**

**Table 5:** Average FR-IQA values of poor-lighted face images processed by our methods trained by various loss items on the MultiPIE dataset.

	VGG-16	Conv1-5	Conv1-6	Conv1-7	Combination
Metrics					
SSIM [53]		0.7762	0.7756	0.7715	**0.7814**
VIF [54]		0.3642	0.3651	0.3612	**0.3685**
FSIM [55]		0.8828	0.8830	0.8819	**0.8837**

## Abbreviations

The following abbreviations are used in this manuscript:

MSFM	Multi-stage Feature Maps
GANs	Generative Adversarial Networks
SSIM	Structural Similarity
VIF	Visual Information Fidelity
FSIM	Feature Similarity
LAD	Least Absolute Deviations
LAE	Least Absolute Errors
FRGC	Face Recognition Grand Challenge
FR-IQA	Full Reference Image Quality Assessment
FAR	False Acceptance Rate
LT	Logarithmic Transform
HE	Histogram Equalization
SQI	Self-quotient Image
LDCT	Logarithmic Discrete Cosine Transform
LTV	Logarithmic Total Variation
LN	Local Normalization
